# Correlation between Duffy blood group phenotype and breast cancer incidence

**DOI:** 10.1186/1471-2407-12-374

**Published:** 2012-08-28

**Authors:** Xiao-feng Liu, Lian-fang Li, Zhou-luo Ou, Rong Shen, Zhi-min Shao

**Affiliations:** 1 Department of Breast, Nanjing Maternity and Child Health Hospital of Nanjing Medical University, Nanjing, 210004, People's Republic of China; 2 Department of Breast surgery, Cancer Hospital/Cancer Institute, Department of Oncology, Fudan University, Shanghai, 200032, People's Republic of China; 3 Department of Breast, Henan Province Tumor Hospital, Zhengzhou, 450008, People's Republic of China; 4 Department of gynaecology/obstetrics, Nanjing Maternity and Child Health Hospital of Nanjing Medical University, Nanjing, 210004, People's Republic of China

**Keywords:** Duffy blood group, Duffy antigen/receptor for chemokines (DARC), Breast cancer

## Abstract

**Background:**

Different ethnicities have different distribution of Duffy blood group (DBG) phenotypes and different breast cancer morbidity. A study in our lab demonstrated that Duffy antigen/receptor for chemokines (DARC, also known as DBGP, the Duffy protein phenotype), led to the inhibition of tumorigenesis. Therefore, we tested the hypothesis that DBGP is correlated with breast cancer occurrence.

**Methods:**

DBGP proteins were examined by indirect antiglobulin testing with anti-FYa and anti-FYb antibodies. The phenotypes were classified into four groups according to the agglutination reactions: FYa + FYb+, FYa + FYb-, FYa-FYb + and FYa-FYb-. The phenotypes and pathological diagnosis of consecutively hospitalized female patients (n = 5,022) suffering from breast cancer at the Shanghai Cancer Hospital and Henan Province Cancer Hospital were investigated. The relationships between DBGP expression with breast cancer occurrence, axillary lymph status, histological subtype, tumor size pathological grade and overall survival were analyzed.

**Results:**

The incidence of breast cancer was significantly different between FYa + FYb + (29.8%), FYa + FYb- (33.2%), FYa-FYb + (45.6%) and FYa-FYb- (59.1%; P = 0.001). Significant different numbers of breast cancer patients had metastases to the axillary lymph nodes in the FYa + FYb + group (25.1%), FYa + FYb- (36.9%), FYa-FYb + (41.0%) and FYa-FYb- (50.0%, (P = 0.005). There was a statistical significance (p = 0.022) of the overall survival difference between patients with difference phenotypes. No significant difference was observed in cancer size (*t*-test, p > 0.05), histological cancer type (Fisher's exact test, p > 0.05) or histological grade (Fisher's exact test, p > 0.05) between every each DBGP group.

**Conclusions:**

DBGP is correlated with breast cancer incidence and axillary lymph node metastasis and overall survival. Further investigations are required to determine the underlying mechanism of Duffy blood group phenotype on breast cancer risk.

## Background

Duffy blood group (DBG) system consists of genotype systems, phenotype systems and five antigens. The genotype system of DBG consists of four alleles, *FYA*, *FYB*, *FYBES*, and *FYBWK*. The DBG phenotype (DBGP) system consists of five phenotypes [FYa + FYb+, FYa + FYb-, FYa-FYb+, FYa-FYb + (wK), and FYa-FYb-]. In addition to these genotype and phenotype systems, the DBG system consists of five antigens (DBGP proteins), termed FYa, FYb, FY3, FY5 and FY6 [1]. The majority of antigens are FYa and FYb, which are encoded by the allelic *FYA* and *FYB* genes. Anti-FYa and anti-FYb antibodies define four red blood cell (RBC) phenotypes: FYa + FYb-, FYa-FYb+, FYa + FYb+, and FYa-FYb- [2].

The DBGP system is embodied by proteins that carry blood group antigens on the surfaces of RBC. These proteins have the same structural and functional basis as Duffy antigen/receptor for chemokines (DARC), which is the chemokine decoy receptor on the surface of RBCs and other cells [3,4]. The DBGP protein on the surfaces of RBCs has the same structure and decoy function as DARC, which was termed DBGP/DARC in this paper.

DBGP/DARC is a 336 amino-acid glycoprotein that can bind to members of the CXC and CC classes of chemokines, including interleukin-8 (IL-8), monocyte chemotactic protein-1 (MCP-1) and RANTES (Regulated on Activation, Normal T Expressed and Secreted) [5,6]. These chemokines have been implicated in the pathogenesis of breast cancer [7-9].

Besides these ligands are correlated with breast cancer, DBGP/DARC has aroused the interest in cancer research as it has been implicated in non-small cell lung cancer tumorigenesis (NSCLC) [10], prostate cancer incidence [11,12], and breast cancer development [13]. NSCLC tumor cells that overexpress DBG have increased levels of tumor necrosis [10]. DBGP/DARC clears angiogenic CXC chemokines and reduced chemotaxis in the vasculature [11,14]. Moreover, DBG interacts with a prostate cancer metastasis suppressor gene, *KAI1*, and inhibits the proliferation of prostate cancer cells [15,16].

Our previous studies have observed that breast cancer lines, MDA-MB-231 and MDA-MB-435, that overexpressed DBGP/DARC induced the inhibition of tumorigenesis through interfering with tumor angiogenesis in rats, and this inhibition was associated with decreased expression levels of CCL2 (Chemokine C-C motif ligand 2, one of DBGP/DARC ligand), decreased microvascular density and decreased MMP-9 (matrix metalloproteinase-9) expression in xenograft tumors [13]. Furthermore, the downregulation of DBG was associated with lymph node metastasis in human breast cancer [17].

Moreover, different ethnicities have different distribution of Duffy blood group (DBG) phenotypes and different breast cancer morbidity.

To date, no epidemiological study has validated the above findings. Based on these previous findings, we designed this study to assess whether there is a correlation between Duffy blood group phenotype (DBGP/DARC) and breast cancer incidence using a clinical epidemiological approach.

## Methods

### Study subjects

This study was carried out on a series of 5,022 consecutively hospitalized female patients (mean age, 50.5 ± 13.1 yr; range, 13–83 yr) with either benign or malignant breast disease. All patients were hospitalized at either the Shanghai Cancer Hospital or Henan Province Cancer Hospital between July 15^th^, 2006 and November 14^th^, 2007. The protocol of this study was approved by the human research committee of both hospitals, and informed consent was obtained from each patient. All patients were followed-up to determine their clinical outcome.

Patients were excluded from the study if they fulfilled one or more of the following criteria: 1) no histological diagnosis was obtained as the patient had not undergone surgery or core biopsy (n = 62); 2) histology revealed a malignant phyllodes tumor (n = 33) and so could not be classified as either a breast cancer or a benign lesion; and 3) patients had undergone neoadjuvent therapy which could have influenced the postoperative histological diagnosis (n = 228). Overall, 323 patients were excluded from the study and 5,022 patients were enrolled in this study.

### Blood samples and test reagents

A 2-ml sample of whole blood was obtained in a glass tube pretreated with EDTA (100 μg/mL), and stored at 4°C. Anti-FYa and Anti-FYb reagents were obtained from Biotest Corporation (Germany). Anti-human globulins were obtained from the Shanghai Blood Center (China), who kindly offered FYa- and FYb-positive or -negative RBCs as a gift.

### Indirect antiglobulin-test

Within 5 days of sampling, blood samples were subjected to the indirect antiglobulin-test according to standard procedures described in the instructions accompanying the reagents and previously published methods [18]. The reaction strength was evaluated according to the Technical Manual, 12^th^ Edition, Section 1, American Association of Blood Banks.

A positive and a negative control were performed in a parallel experiment. Patient phenotypes were classified into four types: FYa + FYb+, FYa + FYb-, FYa-FYb + and FYa-FYb- according to the presence or absence of FYa and FYb antigens.

### Pathological diagnosis

All of the histological diagnosis was based on formalin-fixed paraffin-embedded sections. The pathological features were reviewed by two experienced pathologists who were specialized in breast pathology independently. The chief pathological diagnosis was the determination of whether a lesion was malignant or benign. The histopathological features of breast cancer were reported, including tumor size, histological cancer subtype and pathological grade.

If the benign leision(s) and breast cancer(s) co-exist in one patient, the patient is determined and subjected to statistical analysis as breast cancer patient.

### Statistical analysis

All statistical analyses were performed using SPSS (version17.0). A P-value <0.05 was considered to be statistically significant. Between-group comparisons for the incidence of breast cancer and axillary lymph node metastasis in every DBGP group were performed with the Chi-square test. The significance of overall survival difference among DBGP was tested according to the log rank test. The difference in tumor size between DBGP groups was analyzed by the Student's *t*-test. The differences in histological cancer types and histological grades were compared using the Fisher's exact test.

## Results

### DBGPs distribution (DBGPD)

The details of a total of 5,022 consecutively enrolled patients with 6,012 pathological diagnoses are summarized in Table 1. There were 3,855 benign lesions in 3,060 patients, 1,992 breast cancers in 1,962 patients, and 165 additional benign lesions in the 1,962 patients with breast cancer. Overall, 620 patients were FYa + FYb + (12.3%), 4,262 were FYa + FYb- (84.9%), 120 were FYa-FYb + (2.4%) and 20 were FYa-FYb- (0.4%) (Table 2).

**Table 1:** **The pathological diagnoses in a total of 5,022 female hospitalized patients**^**†**^

**Lesions**	**Patients (total 5022)**
breast cancer	
DCIS (duct carcinoma in situ)	125
invasive lobular breast cancer	59
invasive ductar breast carcinoma	1683
mucinous carcinoma	41
tubular carcinoma	71
unknown types^‡^	13
total^§^	1992
benign	
fibroadenoma	881
intraductal papilloma	265
mastopathia	2809
galactoma	22
mammary myoepitheliosis	23
benign phyllodes tumor	20
total^||^	4020

† 6,012 pathological diagnoses were obtained from 5,022 patients.

‡ Includes 11 cases of supplementary radical operation and two cases of occult breast cancer.

§ 1,992 breast cancers in 1,962 patients; 26 pts out of 1,825 pts had bilateral invasive cancers, three pts had invasive cancers and contralateral DCIS. One patient out of 121 patients with DCIS had bilateral DCIS, 11 cases underwent a supplementary radical operation, and two cases of occult breast cancer.

|| 3,855 breast benign lesions in 3,060 patients; 165 breast benign lesions in the above 1,962 breast cancer patients.

**Table 2:** The numerical value and percentage of breast cancer cases and cancer size in different DBG phenotypes of 5,022 patients (6,012 pathological diagnoses)

**DBGP**	**Breast cancer**	**Benign disease**	**Total**	**Cancer size**	**DBGPF**^†¶^
Fya + Fyb+	219(29.8%1) ^‡^	517(70.2%)	736(100%)	26 ± 9^§^	620(12.3%)
Fya + Fyb-	1698(33.2%)^||^	3420(66.8%)	5118(100%)	26 ± 10	4262(84.9%)
Fya-Fyb+	62(45.6%)	74(54.4%)	136(100%)	27 ± 9	120(2.4%)
Fya-Fyb-	13(59.1%)	9(40.9%)	22(100%)	29 ± 7	20(0.4%)
Total	1992	4020	6012		5022(100%)

^†^ DBGPF = DBGP frequency.

^‡^ Among these 219 breast cancer, 11 were DCIS; one case was a supplementary radical operation.

^§^ Among these 219 breast cancers, one case supplementary radical operation was excluded from statistics of cancer size because of primary breast cancers had been removed.

^||^ Among these 1,698 breast cancers, 112 were DCIS, 10 cases were supplementary radical operations and two cases were occult breast cancers.

^¶^ Among these 1,698 breast cancers, two cases of axillary lymph nodes biopsies were excluded from the statistics regarding cancer size because the presence of occult breast cancer; 10 cases of supplemental radical operations were excluded from the statistics regarding cancer size as the primary breast cancers had already been removed.

### Breast cancer incidence and surgical treatment

Among the 1,992 breast cancers in 1,962 patients, 125 were DCIS and 1,867 were invasive breast cancers. Of these, 26 out of the 1,825 patients had bilateral invasive cancers, and three patients had invasive cancers and contralateral DCIS. One patient out of the 125 patients with DCIS had bilateral DCIS. Eleven patients had an operable locoregional recurrence, and two patients had occult breast cancers.

Among the 1,867 invasive breast cancers, 1,406 patients underwent total mastectomy and axillary dissection at levels I or II (modified radical mastectomy), 294 underwent breast-conserving surgery, 156 underwent sentinel lymph node biopsies, and 11 patients with operable locoregional recurrence underwent radical mastectomy. Among the 125 patients with DCIS, 76 underwent breast-conserving surgery, 35 underwent a total mastectomy, and 14 patients with DCIS and microinvasion underwent sentinel lymph node biopsies.

### The relationship between DBGPD and breast cancer incidence and overall survival

A statistically significant difference was observed between every DBG phenotype and breast cancer incidence (29.8% were FYa + FYb+; 33.2% were FYa + FYb-; 45.6% were FYa-FYb + and 59.1% were FYa-FYb-; P = 0.001; Table 2).

**Figure 1:**
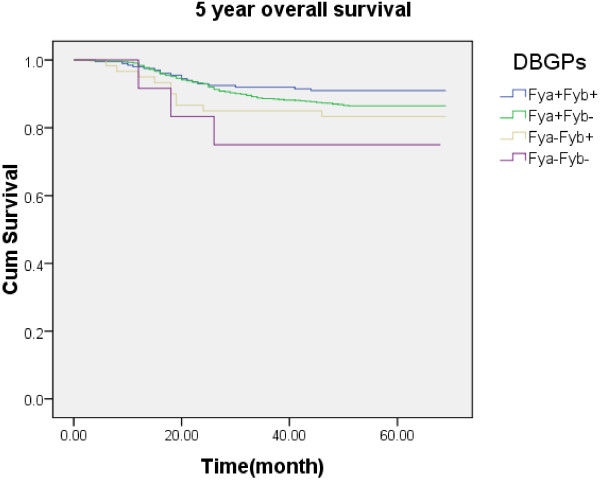
**The 5 year overall survival curves indicated a statistical significance (p = 0.02) of the overall survival difference between patients with difference phenotypes.** The 5 year overall survival distribution was 91.0% in Fya + Fyb+, 86.5% in Fya + Fyb-, 83.3% in Fya-Fyb + and 75.0% in Fya-Fyb-.

There was a statistical significance (p = 0.022) of the overall survival difference between patients with difference phenotypes (Figure 1). The overall survival curves were generated using Kaplan-Meier method.

### The relationship of DBGPD and clinicopathological variables

No significant difference was observed between each DBG phenotype and tumor size (Table 2; p > 0.05). A statistically significant difference in the incidence of axillary lymph node metastasis were observed in breast cancer patients with FYa + FYb + (25.1%), FYa + FYb- (36.9%), FYa-FYb + (41.0%) and FYa-FYb- (50.0%; P = 0.005) phenotypes (Table 3). The difference in pathological grades between every DBG phenotype was not significantly different (p > 0.05; Table 4)*.* There was also no correlation between DBGPs and the histological cancer subtypes (p > 0.05; Table 5). No significant difference was observed between the DBG phenotype and patient age (Student's *t*-test, p > 0.05) or menopausal status (Fisher's Exact Test, p > 0.05; data not shown).

**Table 3:** Axillary lymph node status of 1,867 invasive breast cancers (absolute numbers and percentages)

**DBGPs**	**No metastasis**	**Metastasis**	**Total**
FYa + FYb+	155 (74.9%)	52 (25.1%)	207 (100%)
FYa + FYb-	1002 (63.1%)	585 (36.9%)	1587 (100%)
FYa-FYb+	36 (59.0%)	25 (41.0%)	61 (100%)
FYa-FYb-	6 (50.0%)	6 (50.0%)	12 (100%)
Total	1199 (64.2%)	668 (35.8%)	1867 (100%)

**Table 4:** The pathological grade of 1,867 invasive breast cancers

**DBGPs**	**Grade I**	**Grade II**	**Grade III**	**Total**
FYa + FYb + ^†^	17 (9.0%)	135 (71.8%)	36 (19.1%)	188 (100%)
FYa + FYb-^‡^	105 (7.0%)	1077 (72.2%)	310 (20.8%)	1492 (100%)
FYa-FYb + ^§^ 5	(8.2%)	38 (62.3%)	18 (29.5%)	61 (100%)
FYa-FYb-	1 (7.7%)	9 (69.2%)	3 (23.1%)	13 (100%)
Total	128 (7.3%)	1259 (71.8%)	367 (20.9%)	1754 (100%)

^†^ 18 cases of FYa + FYb + phenotypes had no pathological grades as follows: one case of supplementary radical surgery; three cases mucinous carcinoma; 14 cases of invasive lobular breast cancer.

^‡^ 94 cases of FYa + FYb- phenotypes had not pathological grading as follows: 10 cases of supplementary radical operation; 45 cases of invasive lobular breast cancer; 37 cases of mucinous carcinoma; two cases of occult breast cancer.

^§^ One case of mucinous carcinoma.

**Table 5:** Correlations between the histological type of 1,979 breast cancers and DBGP status (11 cases of supplementary radical surgery and two cases of occult breast cancer were excluded)

**DBGPs**	**DCIS**	**ILBC**^†^	**IDBC**^‡^	**MC**^§^	**TC**^||^	**Total**
FYa + FYb+	11(5.0%)	12(5.5%)	184(84.8%)	3(1.4%)	8 (3.7%)	218(100%)^¶^
FYa + FYb-	112(6.6%)	45(2.7%)	1431(84.9%)	37(2.2%)	61(3.6%)	1686(100%)^††^
FYa-FYb+	1 (1.6%)	1 (1.6%)	57(91.9%)	1(1.6%)	2(3.2%)	62(100%)
FYa-FYb-	1 (7.7%)	1(7.7%)	11(84.6%)	0	0	13(100%)
Total	125	59	1683	41	71	1979

^†^ ILBC = invasive lobular breast cancer.

^‡^ IDBC = invasive ductal breast carcinoma.

^§^ MC = mucinous carcinoma.

^||^ TC = tubular carcinoma.

^¶^ One case of supplementary radical operation.

^††^ 10 cases of supplementary radical surgery and two cases of occult breast cancer were excluded because of the lack of a primary lesion.

## Discussion

### Breast cancer incidence was higher in FYa-FYb + and FYa-FYb-

The results of the current study indicated that breast cancer occurred at significantly higher levels (P = 0.001) in patients with the FYa-FYb + (45.6%) and FYa-FYb- (59.1%) phenotypes than the FYa + FYb + (29.8%) and FYa + FYb- (33.2%) phenotypes. One potential mechanism for this is that the DBG-ligand binding affinity on RBC membranes differs between DBGPs, which may result in different degrees of tumorigenicity.

Tournamille et al. found that a chemokine-binding pocket was defined by the close proximity of the first and fourth transmembrane domains of the DBG/DARC protein and also by the importance of the N-terminal extracellular region correlated to chemokines binding to the DBG protein [19,20]. Woolley et al. developed a flow cytometric method to test the quantity of DBG on the surface of RBCs [21]. They found that FY6 levels were significantly lower on mature RBCs of the FYB/FYB genotype than on those of the FYA/FYA or FYA/FYB genotype. Beside this, 5,000-10,000 DBG molecules were found on a single RBC. Horuk et al. developed saturation binding studies on a erythroleukemic cell line (HEL) and observed that the DBG density was 12,818 +/− 965 binding sites for every cell [5]. These data suggested that RBCs with abundant DBG expression on their surfaces might clear many angiogenic CXC chemokines and decrease chemotaxis in patients with breast cancer. Thereafter, the tumorigenicity of breast cancer cells could be attenuated.

Woolley et al. indicated that Fy6 levels were significantly lower on reticulocytes and mature RBCs of the FYB/FYB genotype (encoding the FYa-FYb + phenotype) than on those of the FYA/FYA (encoding the FYa + FYb- phenotype) or FYA/FYB genotypes (encoding FYa + FYb+) [21]. Moreover, the FYa + FYb + phenotype presented the FYa and FYb antigens, FYa + FYb- presented the FYa antigen had no FYb antigens, and so on. Based on these published reports, we tentatively propose that different DBGPs had different quantities and/or quality of DBG expression on the surface of every RBC. There might be more Duffy antigens expressed on one RBC belonging to an individual with the FYa + FYb + phenotype and a FYa + FYb- individual than on FYa-FYb + or FYa-FYb- individuals. Consequently, FYa + FYb + and FYa + FYb- individuals would have higher chemokine binding capacities than FYa-FYb + and FYa-FYb- individuals, and the FYa + FYb + and FYa + FYb- phenotype offer more protection against breast cancer than the FYa-FYb + and FYa-FYb- phenotypes. Therefore, patients with the former two phenotypes appear to be at a lower risk of breast cancer than patients who express the latter two phenotypes.

### DBGPD and tumor size and axillary lymph nodes status

A previous study had indicated that there was no difference in the frequencies of DBGPs between breast cancer patients compared to a healthy Han population in China (data not shown) [22]. One possible reason for no significant difference in tumor size between each DBGP was that these patients were only diagnosed when their mass was great enough to be discovered. A subclinical period was therefore present before this point.

Significantly more breast cancer patients had axillary lymph node metastases with in FYa-FYb- (50.0%) and FYa-FYb + (41.0%) than that of FYa + FYb- (36.9%) and FYa + FYb + (25.1%), which confirmed our hypothesis. The regular pattern of tumor size as a predictor of axillary node metastases in patients with breast cancer was generally applicable in the clinical practice. However, even patients with small tumours (0-5 mm) have the possibility of axillary involvement (7–14.5%) [23]. The above-described potential mechanism for different DBGPs resulting in different degrees of tumorigenicity might explain this finding.

### Study limitations

Firstly, study subjects were all female patients who were already suffering from breast diseases, and no healthy population was studied prospectively. Therefore, further studies of this protein are required. Secondly, this descriptive and explorative study deduced the mechanism of the link between different DBGPs and breast cancer theoretically, and further studies into the underlying molecular mechanisms are warranted.

## Conclusions

DBGPs have a statistically significant effect on breast cancer incidence and the likelihood of axillary lymph node metastasis.

## Abbreviations

DBG: Duffy Blood Group; DARC: Duffy antigen/receptor for chemokines; DBGP: DBG phenotype; RBC: Red blood cell; DBGPD: DBG phenotypes distribution; DCIS: Ductal carcinoma *in situ*.

## Competing interests

The authors declare that the y have no competing interests.

## Authors’ contributions

XFL carried out the indirect antiglobulin-test and prepared the manuscript. LFL performed the clinical data analyses and statistical analyses and helped to prepare the manuscript. ZLO participated in the design of the study. RS conceived of the study and gave administrative support for the manuscript. ZMS conceived of the study and gave financial support for the manuscript. All authors read and approved the final manuscript.

## Pre-publication history

The pre-publication history for this paper can be accessed here:

http://www.biomedcentral.com/1471-2407/12/374/prepub
